# Whey peptide-based enteral diet attenuated elastase-induced emphysema with increase in short chain fatty acids in mice

**DOI:** 10.1186/s12890-015-0059-2

**Published:** 2015-06-10

**Authors:** Koichi Tomoda, Kaoru Kubo, Kazuo Dairiki, Taketo Yamaji, Yoshifumi Yamamoto, Yasue Nishii, Atsuhiro Nakamura, Masanori Yoshikawa, Kaoru Hamada, Hiroshi Kimura

**Affiliations:** Second Department of Internal Medicine, Nara Medical University, 840 Shijocho, , 634-8521 Kashihara, Nara Japan; Laboratory Animal Research Center, Nara Medical University, 840 Shijocho, , 634-8521 Kashihara, Nara Japan; Food Science Research Labs., R&D Div., Meiji Co. Ltd., 540 Naruda, , 250-0862 Odawara, Kanagawa Japan; Faculty of Health Science, Kio University, 4-2-2 Umami-naka, Koryo-cho, Kitakatsuragi-gun,, 635-0832 Nara, Japan

**Keywords:** Elastase, Emphysema, Whey peptide, C57BL/6 mice, Chronic obstructive pulmonary disease, Short chain fatty acids, Cecum

## Abstract

**Background:**

Systemic inflammation is present in chronic obstructive pulmonary disease (COPD). A whey peptide-based enteral diet reduce inflammation in patients with COPD, but its effect on COPD development has not been determined. On the other hand, it is known that short chain fatty acids (SCFAs), which are produced by micro-flora in the gut, attenuates bronchial asthma in mice model.

**Methods:**

Mice with elastase-induced emphysema were fed with 1 of 3 diets (control diet, whey peptide-based enteral diet, or standard enteral diet) to determine the effects of whey peptide-based enteral diet on emphysema and on cecal SCFAs.

**Results:**

The whey peptide-based enteral diet group exhibited fewer emphysematous changes; significantly lower total cell counts in bronchoalveolar lavage fluid (BALF); and significantly higher cecal SCFA levels than either the control or standard enteral diet groups. The total cell count was inversely correlated with total cecal SCFA levels in these three diet groups.

**Conclusions:**

The whey peptide-based enteral diet attenuates elastase-induced emphysema through the suppression of inflammation in the lung. This may be related to the increase in cecal SCFA.

## Background

Chronic obstructive pulmonary disease (COPD), which includes emphysema and chronic bronchitis, is recognized as a major public health issue [1] and involves more than just the airway [2, 3]. Systemic inflammation is present in all disease stages [2] and contributes to comorbidities such as cardiovascular disease, malnutrition, and osteoporosis [2, 4].

A whey peptide-based enteral diet, as well as whey protein and its hydrolysate, has demonstrated anti-inflammatory effects in a variety of experimental animal models [5–8] and COPD patients [9]. However, the effect of a whey peptide-based enteral diet on the development of COPD has not been elucidated. Moreover, the mechanisms explaining how this diet suppresses inflammation have not been fully investigated.

Recent epidemiological studies have demonstrated that dietary fiber intake is related to better lung function and prevention of COPD [10–12]. Dietary fiber is delivered intact to the large intestine and is then metabolized to organic acids by intestinal micro-flora. A selection of short chain fatty acids (SCFAs) present in these organic acids provide benefits for the host [13]. Recently the SCFA has been proved to relate with attenuation of bronchial hypersensitibity in mice model [14]. We recently demonstrated that changes in SCFA levels in the cecum of rats were related to decreased antioxidant capacity during cigarette smoke exposure; therefore, these changes may contribute to the regulation of lung inflammation [15].

The current study aimed at investigating the ability of a whey peptide-based enteral diet to suppress elastase-induced emphysema in mice and to alter the SCFA levels in the cecum. Moreover in order to explore the relationship between the gut environment and the lung under instillation of elastase, the correlation between the changes in cell counts in the lung and alteration in the SCFA levels in elastase- instilled mice was investigated.

## Methods

### Animals and diets

Six-week-old, female C57BL/6 mice were purchased from Japan SLC, Inc. (Shizuoka, Japan) for use in this study. Animals were preconditioned with a commercial solid diet (AIN-93G; Oriental Yeast Co., Ltd., Tokyo, Japan) and water *ad libitum* for 2 weeks before instillation of elastase in the laboratory animal research center at Nara Medical University. Following this preconditioning, the animals were divided into 3 groups according to dietary composition: (1) control diet (AIN-93G; n = 4); (2) lyophilized enteral diet, namely, a whey peptide-based enteral diet (MEIN®; Meiji Co., Ltd., Tokyo, Japan; n = 4); and (3) standard enteral diet (MEIBALANCE®; Meiji Co., Ltd., Tokyo, Japan; n = 4) (Table 1). Each group was fed the respective diet for the 4 weeks of the study.

**Table 1:** The composition of experimental diets (per 100 kcal)

Composition	Control diet (AIN-93G)	Whey peptide-based enteral diet	Standard enteral diet
Proteins (g)	4.8	5.0	4
Proteins (%kcaI)	19	20	16
Protein Sources	case in	Whey peptides	Milk protein
	L-cystine	fermented milk	Na case inate
Carbohydrates (g)	16	14.5	15.5
Carbohydrates (%kcal)	64	55	59
CHO Sources	cornstarch	isomaltulose	dextrin
	sucrose	dextrin	
Lipids (g)	1.9	2.8	2.8
Lipids(%kcal)	17	25	25
MCT (g)	-	0.59	-
EPA,DHA (g)	-	0.06	-
n-6/n3	-	2.0	3.2
Vitamins			
Vitamin A (μg RE)	32	150	60
Vitamin D (μg)	0.70	0.75	0.50
Vitamin E (mg)	2.0	5.0	3.0
Vitamin K (μg)	24	3.4	3.1
Vitamin B1 (nm)	0.13	0.25	0.15
Vitamin B2 (mg)	0.16	0.30	0.20
Niacin(mg)	0.8	3.0	1.6
Vitamin B6(mg)	0.16	0.30	0.30
Vitamin B12(μg)	(166	(160	(160
Folic acid (μg)	50	50	50
Biotin (μg)	5.3	7.5	15
Vitamin C (mg)	-	50	16
Choline (mg)	28	9.2	1.7
Carnitine (mg)	-	15	-
Minerals			
Sodium(mg)	28	70	110
Potassium (mg)	80	80	100
Calcium (ne)	133	80	60
Magnesium (mg)	14	20	3)
Phosphorus (mg)	80	70	60
Iron (mg)	1.2	1.0	1.0
Zinc (mg)	1.0	1.0	0.80
Copper(mg)	0.160	0.050	0.080
Manganese (mg)	0.266	0.175	0.200
Chromium (μg)	26.6	2.96	3.00
Molybdenum (μg)	4.0	2.5	2.5
Selenium(μg)	4.8	5.0	3.5
Iodine (μg)	5.3	9.7	15
Chloride (mg)	43	80	140

MCT: median chain triglyceride, EPA: eicosapentaenoic acid, DHA: docosahexaenoic acid

RE: retinol equivalent. (−): No additives

The animals were kept in a limited-access barrier housing, which was maintained at room temperature (22 ± 1 °C), a humidity level of 55 ± 10 %, and a 12-h light/dark cycle with illumination extending from 08:00 to 20:00. All procedures were carried out under the control of our committee in accordance with The Guidelines for Animal Experiments in the Nara Medical University and Guiding Principles for the Care and Use of Laboratory Animals approved by The Japanese Pharmacological Society.

### Elastase emphysema model

We used an elastase-induced emphysema model, based on previously published protocol [16]. After the preconditioning, 1.2 UI/body porcine pancreatic elastase (PPE), freshly prepared in phosphate buffered saline (PBS), was administered intratracheally at 50 μL directly into the airways under pentobarbital sodium anesthesia (Somnopentyl®, 50 mg/kg, i.p.; Kyoritsu Seiyaku Co., Ltd., Tokyo, Japan). PBS-instilled mice fed with the control diet were used as a sham [15] group.

### Analyses of BALF and morphology

We analyzed BALF and morphology as previously reported [16]. Four weeks after PPE instillation, the animals received an intraperitoneal injection of pentobarbital sodium before euthanization. After removal of both lungs, 1 mL of sterile PBS was instilled and then collected twice using a 1-mL syringe. The collected lavage fluid was analyzed as BALF, and the cells were counted using a hemocytometer. After collecting the cells supernatant of the BALF was stored at −80 °C until measurement of inflammatory cytokines. Cell differentials were counted using smears prepared by Cytospin and stained with Diff-Quik (Sysmex, Kobe, Japan). Whole lungs were inflated with 4 % paraformaldehyde at a pressure of 25 cm H_2_O and then fixed in formalin for 24 h, embedded with paraffin, sectioned in the sagittal plane, and stained with hematoxylin and eosin [16].

The mean linear intercept (MLI) as the morphologic parameter on the lung tissues was calculated as follows, based on previously published protocols [17]. Digital images were captured at both 200× and 400× magnifications. Horizontal, vertical and diagonal grid lines were overlaid and used to count the number of alveolar septa intersections. MLI was calculated as follows: length of grid lines divided by the number of intersections with alveolar septa.

### Detection of SCFAs in the cecal content

We measured SCFA levels in the cecum as previously reported [18]. The ileocecal and cecocolonic junctions were ligated, and the cecum was removed. The cecum was immediately frozen and stored at −80 °C until further analyses. The cecal contents were drained from the cecocolonic junction into a 50-mL vial and then used for analyses. SCFAs in the cecum were measured by high performance liquid chromatography (Shimadzu, Kyoto, Japan) with an internal standard. Approximately 100 mg of the cecal content was homogenized using ultrasonication in 1 mL of a 10 mM sodium hydroxide aqueous solution containing 5 mM crotonic acid as an internal standard, and the mixture was centrifuged at 10,000 × *g* for 15 min. The fat-soluble substances in the supernatant were extracted using chloroform. The neutralization of the cecal contents with sodium hydroxide prevented the extraction of SCFAs and crotonic acid by the chloroform. The aqueous phase was then passed through a membrane filter (cellulose acetate, 0.20 μm pore size; DISMIC-13cp; Advantec Toyo, Tokyo, Japan). The sample was applied to a high performance liquid chromatography column (Shimadzu, Kyoto, Japan) for analysis of SCFAs.

SCFAs were measured according to the method of Hoshi et al. [19] with a minor modification. SCFAs were separated in an ion-exclusion column and detected by a post-column pH-buffered electroconductivity detection method, using a double-connected H-type cation exchanger column (Shim-pack SCR-^1^02H, 8 mm i.d. ×30 cm long; Shimadzu, Tokyo, Japan), with a column temperature of 45 °C, a mobile phase of a 5 mM p-toluene sulfonic acid aqueous solution (0.8 mL/min flow rate, 45 °C), an electroconductivity detector of positive polarity at 45 °C (type CDD-6A; Shimadzu, Tokyo, Japan), and a detection reagent of a 20 mM bis Tris aqueous solution containing 5 mM p-toluene sulfonic acid and 100 μM ethylenediaminetetraacetic acid (0.8 mL/min flow rate, 45 °C).

Each cecal SCFA level is adjusted for weight of the cecal contents and indicated as μmol/g cecal content. The total SCFA level was calculated as the sum of acetate, propionate, n-butyrate, i-butyrate, and i-valerate.

### Detection of inflammatory cytokines in serum

Blood samples were obtained from heart after euthanization. The blood was centrifuged at 4 °C and the plasma was collected and stored at −80 °C until measurement of inflammatory cytokines. The inflammatory cytokines (TNF-α and IL-6) levels in the plasma and the BALF were determined using a mouse inflammation cytometric bead array kit (CBA; BD Biosciences, San Diego, CA, USA). The assay sensitivity of TNF-α and IL-6 was 7 pg/μL, and 5 pg/μL, respectively.

### Relationship between total cecal SCFA content and total cell count in the BALF from elastase-instilled mice

To investigate the relationship the gut environment and the lung after elastase-instillation, we investigated relationship between total cecal SCFA levels and total cell count in the BALF from elastase-instilled mice.

### Statistical analysis

Comparisons between the 2 groups were conducted using Mann–Whitney *U* tests. Relationship between total cecal SCFA content and total cell count in the BALF from elastase-instilled mice was conducted using single linear regression analysis. A *p* value <0.05 was considered statistically significant.

## Results

### Changes in body weight

**Fig. 1:**
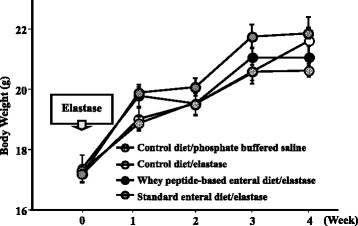
Changes in body weight in the 4 weeks following elastase instillation to induce emphysema. Results are expressed as mean ± standard error of mean. Symbols:, phosphate buffered saline-instilled mice with control diet (sham; n = 4);, elastase-instilled mice on the control diet (n = 4);,elastase-instilled mice on the whey peptide-based enteral diet (n = 4);, elastase-instilled mice on the standard enteral diet (n = 4). There were no significant differences at any weeks before and after elastase instillation between the 4 groups, compared using Mann–Whitney *U* tests

Figure 1 illustrates the changes in body weight that occurred over the course of the experiment. There were no significant differences among the 4 groups in body weight at the any week before and after elastase instillation (sham group and elastase-induced groups [control, whey peptide-based enteral, and standard enteral diets]).

### Whey diet mitigated elastase-generated emphysema like phenotype

**Fig. 2:**
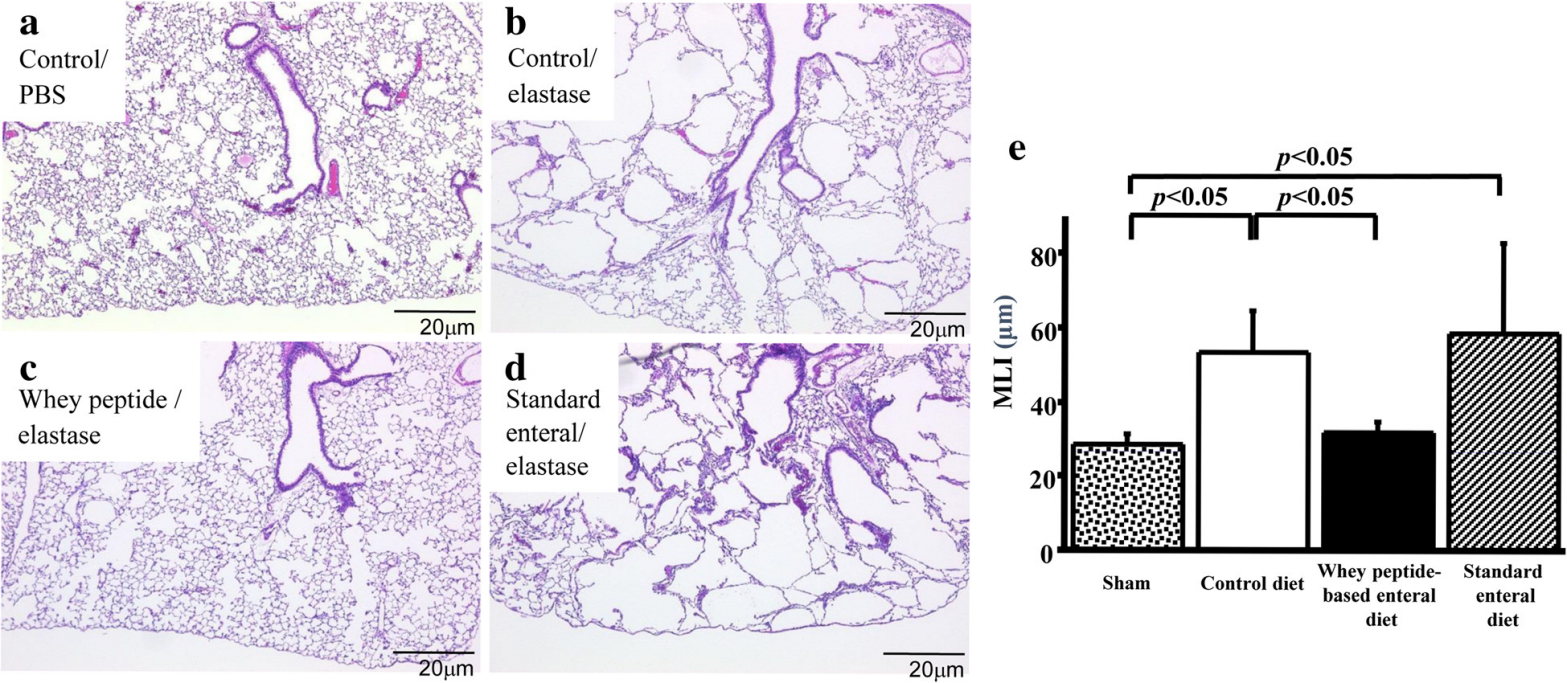
Whey peptide suppressed elastase-induced emphysematous changes. Histologically in the control diet groups, elastase-instillation induced emphysematous lesions with enlargement and disruption of alveolar walls (**a** and **b**). These changes were remarkably suppressed by whey peptide (**c**), while these were not suppressed by standard enteral diet (**d**). Changes in mean linear intercept (MLI), one of the morphologic parameters, were shown at **e**. In the control diet groups these were enlarged by elastase-instillation. The enlargements were suppressed by the whey peptide, while those were not suppressed by the standard enteral diet. Results are expressed as mean ± standard deviation of mean and compared using Mann–Whitney *U* tests. The sham group consisted of phosphate buffered saline-instilled mice on a control diet

The morphological differences in the lungs at the end of the 4-week study period are illustrated in Fig. 2. In the control diet group (Fig. 2a), the elastase instillation induced emphysematous lesions with airspace enlargement and destruction of the alveolar wall. In the whey peptide-based enteral diet group (Fig. 2c), both the airspace enlargement and destruction of the alveolar wall were attenuated. The standard enteral diet group (Fig. 2d) did not suppress the airspace enlargement and destruction of the alveolar wall. Comparisons of MLI were shown at Fig. 2e. MLI in the control diet group was significantly increased by elastase instillation (28.7 ± 2.7 μm vs. 53.2 ± 11.4 μm, *P* = 0.021). The increase was significantly suppressed by the whey peptide-based enteral diet (53.2 ± 11.4 μm vs. 31.9 ± 3.1 μm, *P* = 0.021) while the increase was not changed by standard enteral diet.

### Whey diet decreased total cell counts in BALF

**Fig. 3:**
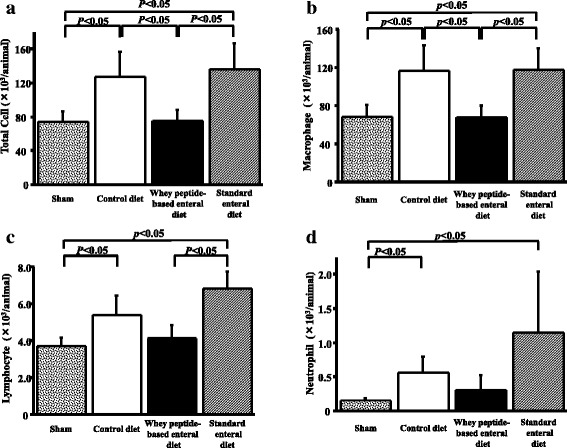
Whey peptide suppressed the increase in total cell counts of BALF. In the control diet groups elastase instillation increased total cell and macrophage counts in the BALF. The increases were significantly suppressed by whey peptide, while these were not suppressed by standard enteral diet (**a** and **b**). In the control diet groups elastase instillation increased lymphocyte and neutrophil counts. The significant increases were similarly observed in the standard enteral diet group, while the increases were not proved in the whey peptide diet group (**c** and **d**). Results are expressed as mean ± standard deviation of mean and compared using Mann–Whitney *U* tests. The sham group consisted of phosphate buffered saline-instilled mice on a control diet

In the control diet group elastase instillation significantly increased not only the total cell count (73.8 ± 12.5 × 10^3^ vs. 127.5 ± 29.0 × 10^3^, *P* = 0.021), but also macrophage (68.5 ± 12.6 × 10^3^ vs. 116.5 ± 27.1 × 10^3^, *P* = 0.021), lymphocyte (3.69 ± 0.47 × 10^3^ vs. 5.39 ± 1.07 × 10^3^, *P* = 0.021) and neutrophil (0.155 ± 0.033 × 10^3^ vs. 0.562 ± 0.236 × 10^3^, *P* = 0.021) counts in the BALF (Fig. 3).

The increases in total cell and macrophage count were significantly suppressed by whey peptide-based enteral diet (127.5 ± 29.0 × 10^3^ vs. 75.0 ± 12.9× 10^3^, *P* = 0.021, 116.5 ± 27.1 × 10^3^ vs. 67.8 ± 12.3 × 10^3^, *P* = 0.021, respectively) while those were not changed by standard enteral diet (Fig. 3A and B). The increases in lymphocyte and neutrophil counts were not proved in the whey diet group (5.39 ± 1.07 × 10^3^ vs. 4.15 ± 0.67 × 10^3^, *P* = 0.149, 0.562 ± 0.236 × 10^3^ vs. 0.309 ± 0.220 × 10^3^, *P* = 0.149, respectively), while those increases were not changed by standard enteral diet (Fig. 3c and d).

### Whey diet increased cecal SCFA levels

**Fig. 4:**
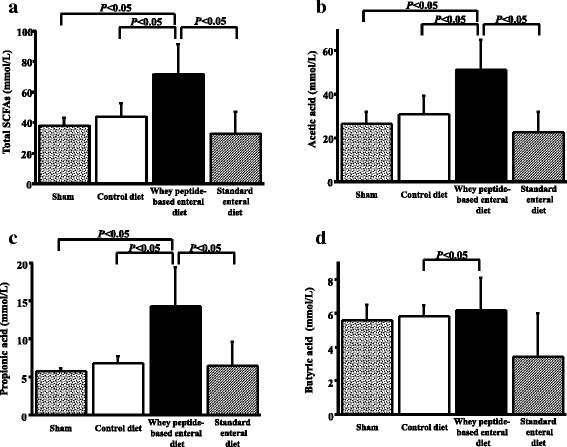
Whey peptide increased short fatty chain acid levels. In elastase-instilled mice whey peptide increased total levels of SCFA (**a**), acetic acid (**b**), propionic acid (**c**) and butyric acid (**d**) while the increases were not proved by standard enteral diet. Results are expressed as mean ± standard deviation of mean and compared using Mann–Whitney *U* tests. The sham group consisted of phosphate buffered saline-instilled mice on a control diet

In the elastase-instilled mice, the whey peptide-based enteral diet increased the total cecal SCFA levels (43.69 ± 9.17 mmol/L vs. 71.69 ± 19.96 mmol/L, *P* = 0.021) (*P* < 0.05). However, there was no significant difference of the content between in the standard enteral diet and control diet groups (Fig. 4a).

The comparison in acetic acid, propionic acid and butyric acid in the SCFAs were shown at Fig. 4b, c and d respectively. Whey peptide-based enteral diet increased the amounts in acetic acid (31.08 ± 8.25 mmol/L vs. 51.27 ± 13.84 mmol/L, *P* = 0.021) and propionic acid level (6.81 ± 0.88 mmol/L vs. 14.23 ± 5.09 mmol/L, *P* = 0.021). However, there was no significant difference in the levels between the standard enteral diet and control diet groups (Fig. 4b and c). The butyric acid level in the whey peptide group was significantly higher than that in control diet group (6.19 ± 1.94 mmol/L vs. 5.80 ± 0.66 mmol/L, *P* = 0.021) (Fig. 4d).

### Whey Inflammatory cytokine levels in plasma and BALF

In plasma or BALF, TNF-α and IL-6 was not detected in any animals.

### Relationship between total cecal SCFA levels and total cell count in the BALF from elastase-instilled mice

**Fig. 5:**
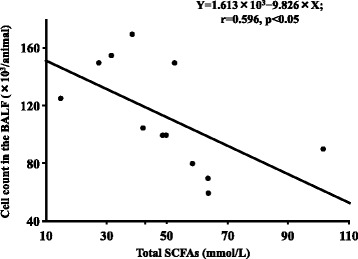
Total cell count in the BALF was inversely correlated with total cecal SCFA levels. Total cell count in the BALF from elastase-instilled mice was inversely correlated with total cecal SCFA levels

Total cell count in the BALF from elastase-instilled mice was inversely correlated with total cecal SCFA levels (r = 0.596, *P* = 0.041) (Fig. 5). This result suggested that whey peptide-based enteral diet accelerated fermentation by micro-flora in the gut and that the acceleration may be related to suppression in the increased total cell counts in BALF.

## Discussion

The present study demonstrated that a whey peptide-based enteral diet suppressed elastase-induced emphysema in mice, compared with both a control diet and a standard enteral diet. The increase in total cell, neutrophil, and lymphocyte counts observed in the lungs of the mice fed with the control diet was not observed in mice with the whey peptide-based enteral diet, suggesting that the resulting anti-inflammatory effect in the lungs may contribute to the suppression of the elastase-induced emphysema.

Systemic inflammation is present in COPD [2, 4], and malnutrition, as one of the systemic effects of COPD, is an independent prognostic factor of disease outcome. Various nutritional therapies have been investigated to improve the prognosis of COPD, and a whey peptide-based enteral diet may suppress the systemic inflammation present in COPD patients. Previous reports have also indicated that a whey peptide-based enteral diet is useful for atopic dermatitis, hepatitis, and gut ischemia reperfusion injury in animal models [5–8]. This may be related to the systemic anti-inflammatory effects provided by the whey peptide-based enteral diet, but this mechanism is not fully understood. In the current study, plasma TNF-α, IL-6 were not detected in the control diet and the whey peptide-based enteral diet groups, indicating that systemic anti-inflammatory effects do not necessarily explain changes in the inflammatory environment in the lungs.

We inferred from recent epidemiological data demonstrating beneficial effects of dietary fiber for lung function and COPD [10–12], together with the importance of dietary fiber in maintaining the gut environment [13], and that changes in the gut environment may contribute to the development of COPD. Intestinal micro-flora produce organic acids, including SCFA, which confer anti-inflammatory effects to the host. Our previous results indicated that cigarette smoke alters cecal organic acid levels, and a fiber-free diet, combined with cigarette smoke, decreases the antioxidant capacity related to the gut acid levels. These results led us to further explore the effects of intestinal organic acids on inflammation in other body systems. In this study the use of a whey peptide-based enteral diet increased the SCFA levels of the cecum which in turn significantly correlated with total cell count in the BALF. These results suggest that an increase in intestinal SCFAs may contribute to anti-inflammatory processes in the lung. Not only organic acids but also microflora is crucial in maintaining the gut environment. Analysis of the proportion of microflora needs to be investigated. Further investigation is needed to understand the link between the lung and the gut when consuming a whey peptide-based enteral diet.

## Conclusions

A whey peptide-based enteral diet inhibited elastase-induced emphysema in mice through the suppression of inflammation in the lungs. This may be related to the increase in cecal SCFAs that was present with the diet. Therefore, the results from the study suggest that a whey peptide-based enteral diet may be useful not only for systemic inflammation but also to slow the progression of COPD.

## Abbreviations

BALF: Bronchoalveolar lavage fluid
COPD: Chronic obstructive pulmonary disease
PBS: Phosphate buffered saline
PPE: Porcine pancreatic elastase
SCFA: Short chain fatty acid
